# In-Flight Performance of the ICON EUV Spectrograph

**DOI:** 10.1007/s11214-023-00963-1

**Published:** 2023-03-28

**Authors:** Eric J. Korpela, Martin M. Sirk, Jerry Edelstein, Jason B. McPhate, Richard M. Tuminello, Andrew W. Stephan, Scott L. England, Thomas J. Immel

**Affiliations:** 1 Space Sciences Laboratory, University of California, Berkeley, CA USA; 2 Aerospace & Ocean Engineering, Virginia Tech, Blacksburg, VA USA; 3 U.S. Naval Research Laboratory, Washington D.C., USA

**Keywords:** Extreme ultraviolet, Instrumentation, Ionosphere, Spectrograph

## Abstract

We present in-flight performance measurements of the Ionospheric Connection Explorer EUV spectrometer, *ICON EUV*, a wide field ($17^{\circ} \times 12^{\circ} $) extreme ultraviolet (EUV) imaging spectrograph designed to observe the lower ionosphere at tangent altitudes between 100 and 500 km. The primary targets of the spectrometer, which has a spectral range of 54–88 nm, are the Oii emission lines at 61.6 nmand 83.4 nm. In flight calibration and performance measurement has shown that the instrument has met all of the science performance requirements. We discuss the observed and expected changes in the instrument performance due to microchannel plate charge depletion, and how these changes were tracked over the first two years of flight. This paper shows raw data products from this instrument. A parallel paper (Stephan et al. in Space Sci. Rev. 218:63, 2022) in this volume discusses the use of these raw products to determine O^+^ density profiles versus altitude.

## Introduction

### *ICON* Mission

The *ICON* observatory is a NASA Explorer mission that studies the boundary between Earth and space to understand the physical connection between the atmosphere and outer space. This connection is made in the ionosphere, long known to respond to space weather driven by the sun. Studies in the last half-century have revealed that energy and momentum of our atmosphere have effects of similar magnitude on the ionosphere. *ICON*’s goal is to measure the relative impacts of these drivers.

*ICON* observes the ionosphere from 575 km × 605 km orbit at an inclination of 27 degrees to the equator, employing a suite of four instruments. This orbit results in a precession period of 48 days, which mean for analysis requiring maximum local time-latitude coverage, 48 days of data is required. This precession is small enough that seasonal variations can be well determined. An orbital inclination of $27^{\circ}$ allows rapid access via precession to all available low and middle latitudes, longitudes, and local time. Emission line features in the EUV and FUV are measured by two co-aligned spectrographs to yield ion density altitude profiles. Neutral winds and temperatures are determined with an interferometer working at visible wavelengths, while ion velocities at the observatory are measured with an ion drift meter. A detailed overview of the *ICON* mission was presented by Immel et al. (2018). The *ICON EUV* spectrometer was described in detail in Sirk et al. (2017). The other instruments were described in several papers in the same volume (Englert et al. 2017; Heelis et al. 2017; Mende et al. 2017). Papers utilizing these data and the associated altitude profiles include (Stephan et al. 2022; Tuminello et al. 2022; Wautelet et al. 2022).

### *ICON EUV*

The existence of EUV emission from singly ionized oxygen (O^+^) in the ionosphere of the Earth has long been known and is a useful diagnostic of the ionization state and density of the lower ionosphere (Feldman et al. 1981). The brightest of the Oii dayglow line complexes in the EUV is the 83.4 nmresonance triplet resulting from transition from the $2p^{4}$ $^{4}P$ excited states to the $2p^{3}$ $^{4}S^{0}$ ground state.^1^ The population of ions in the ground state typically peaks at densities on the order of $10^{4}-10^{6}$ cm^−3^, enhancing the probability that an emitted photon from this transition will be reabsorbed resulting in an optical depth to the ICON EUV on the order of 1-10 in this transition. This can make it difficult to disentangle optical depth effects from illumination and ion density effects when attempting to determine density of the O^+^ ion. The nearby triplet at 61.6 nmfrom the $3s$ $^{2}P$ state to the $2p^{3}$ $^{2}D^{0}$ state is optically thin. The two taken together can be used to more directly obtain the ion density and illumination source function than the 83.4 nmemission alone (Stephan et al. 2012). Similar transitions at 53.8 nm, 67.3 nmand 71.8 nmcould theoretically be used to supplement this analysis.

**Fig. 1:**
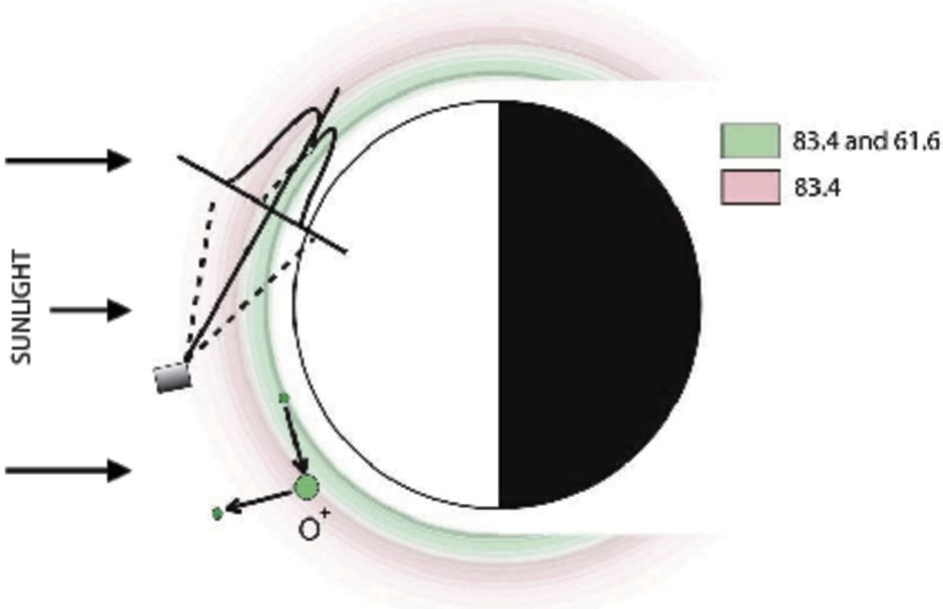
Illustration of the *ICON EUV* observing geometry. Space craft motion is towards the reader. The vertical (imaging) field of view is 17$\,.\!\!\!^{\circ} $3 and is demarcated by dashed lines. The horizontal (spectral) field of view is 12$\,.\!\!\!^{\circ} $1 (plus 0$\,.\!\!\!^{\circ} $76 caused by 12 s of space craft motion). The height of the atmosphere is exaggerated by 5x. Below about 100 km altitude the atmosphere is too dense to hold a significant O^+^ density and too opaque to transmit significant O^+^ emission. The gap in the emission toward the night side represent the shadow of the earth and atmospheric absorption, where O^+^ emission is significantly more faint than the daytime levels. The green circles and arrows are a symbolic representation of ionization and recombination processes of oxygen, and do not represent atmospheric structures. Figure after (Sirk et al. 2017)

The *ICON EUV* spectrometer is designed to measure wide field altitude profiles of the region surrounding the peak O^+^ densities in the lower ionosphere, at altitudes between 100 and 500 km with a vertical resolution of 20 km, and a horizontal resolution of 500 km (Fig. 1. See also Fig. 16 of Immel et al. (2018)). The primary design requirements relate to obtaining the sensitivity and angular resolution necessary to determine the maximum ion density of the F2 layer and the altitude of the maximum density using the 61.6 nmand 83.4 nmemission, while rejecting interference from scattered Hi Ly $\alpha$ and the nearby Hei 58.4 nmline.

In normal observing mode, the *ICON EUV* field of view faces perpendicular to the spacecraft velocity vector and downward to cover this targeted altitude range. Each 12 second exposure images a $12^{\circ}$ wide (spectral) by $17^{\circ}$ high (imaging) wedge of the atmosphere from which daytime ion density altitude profiles are determined. The spacecraft velocity along the orbital track during a 12 s observation results in a spacecraft motion of about 100 km, which is small compared to the 500 km range of the $12^{\circ}$ field of view at the tangent point. In effect each exposure is a snapshot taken at a specific spacecraft position and time. The required sensitivities (3$\sigma$ Minimum Measurable Flux (MMF)) of 7.4 Rayleigh at 61.6 nm and 30 Rayleigh at 83.4 nm were determined based on simulated model inversions of the altitude profiles to derive the O^+^ density versus altitude (Stephan 2016; Stephan et al. 2017). These minimum sensitivities are ∼10% of the maximum ionospheric column density emission. End of life requirements are based upon pre-flight calculations of the worst case sensitivity losses in the microchannel plate detector and continuous low-rate deposition of hydrocarbon contamination on the instrument diffraction grating. Total end of life efficiency loss under these assumptions was 60% from original conditions on receipt of the optics and microchannel plates. Actual degradation has been tracked through on-orbit calibration activities.

## Brief Description of Instrument

For the readers benefit we provide a brief description of the instrument. The detailed design can be found in Sirk et al. (2017). The *ICON EUV* instrument is a imaging spectrograph for observation of wide angle (diffuse) emission consisting of an entrance aperture, a diffraction grating, and a microchannel plate (MCP) detector. The holographic grating is coated with a 40 nm thick coating of Cr overlayed with additional layers of Ir (20 nm) and B_4_C (10 nm) to enhance EUV reflectivity. The MCP detector and spectrograph were both designed and assembled at the Space Sciences Laboratory (SSL) at the University of California Berkeley Campus. The spectrograph is housed in a hermetic vacuum cavity to reduce the possibility of pre-launch contamination. On the ground the instrument was either maintained at vacuum or purged with dry nitrogen to prevent contamination or damage. A sealed one-shot door in front of the instrument was opened in orbit at the start of the mission to allow light to enter.

During operation EUV radiation from the sky enters the 0.90 by 40.0 mm slit, illuminates the 50 by 95 mm toroidal figure, ion-blazed (lamellar profile), holographically ruled diffraction grating (Liard 2015), and is then focused by the grating onto a 19 by 54 mm cross delay line MCP detector with a spatial resolution of 90 μm in the spectral direction, and 160 μm in the imaging direction (Davis et al. 2011). On-board electronics digitize the photon events into an image 169 pixels wide in the spectral dimension, and 108 pixels tall in the imaging dimension. The detector electronics contain a stimulation pulser (aka stim-pulser) that provides a pulse at the low and high delay ends of each of the crossed delay line anodes ∼10 times per second. This provides a fiducial position that can be used to scale the image to correct for temperature related changes in the delay of the anode or electronics. In flight these changes have been very small relative to the coarse resolution of a 169×108 image. The high rate also allows us to calibrate our electronics dead time losses, as deadtime will equally affect the stim events. Anode and electronic distortions as measured on the ground are small compared to the size of our relatively coarse pixels. The differential nonlinearities due to primarily to switching of the time to analog portion of the electronics do appear as a wavelike pattern in the flat field measurement, and as such are removed by flat field correction.

**Fig. 2:**
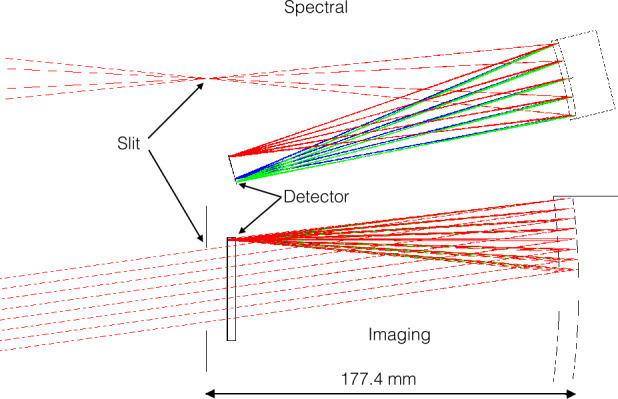
Schematic diagram showing how light of three wavelengths (58.4, 61.6, and 83.4 nm, green, blue, and red, respectively) from the entire $12^{\circ}$ wide spectral field of view is focused onto the detector along the dispersion direction (top), and how collimated, in-band light from a particular altitude angle is focused onto one row on the detector in the imaging direction (bottom). Slit width is exaggerated by a factor of 2 in the top panel. Figure after (Sirk et al. 2017)

The field of view is determined by internal and external baffles, the slit position and the grating dimensions. The optical scheme provides imaging without using a separate telescope optic. In the spectral direction, the toroidal grating focuses an image of the slit onto the detector while in the imaging direction the toroid focuses at infinity resulting in a spectral image where each row is a spectrum from a horizontal slice of the sky $12^{\circ}$ wide, and each column a vertical angular radiance profile at a given wavelength. A diagram of the focusing properties of the grating is shown as Fig. 2. The grating is coated with a special EUV-optimized, low stress B_4_C/Ir/Cr three-layer coating (Windt 2015, priv. commun.).

As would be expected for spherically symmetric emission, for limb observations it is presumed that the majority of the observed emission results from emission near the tangent point. Because the distance to the tangent point depends upon the altitude of the tangent point and the altitude of the spacecraft, for fixed sized pixels a single angular scale cannot be used. The pixel scale varies with altitude from 7.7 km/pixel at 100 km to 3.8 km/pixel at 500 km, with 6.5 km/pixel being typical near the emission peak. The range from 100 to 500 km typically covers 71 pixels.

## The Measurement Process and Necessary Calibrations

The flux in Rayleighs in a vertical bin (pixel) integrated over an observation is

$$ I(\phi)[R] = \frac{4\pi}{10^{6}} \frac{F_{\lambda }(\phi,t)}{\epsilon(\lambda,t) A_{\mathrm{slit}} \Omega(\phi)} \left( \frac{{n(\phi,t)} - b(\phi,t) \frac{F_{b}(\phi ,t)}{F_{\lambda}(\phi,t)}}{t_{\mathrm{exp}}\ d(t)} \right) $$

where

$\epsilon\left(\lambda,t\right )$
is the instrument responsivity averaged over the vertical field of view (also called quantum efficiency) in terms of detector counts generated per incoming photon determined through the combination of ground calibrations and the lunar calibrations and interpolated to the time of the observations.
$A_{\mathrm{slit}}$
is the geometric area of the instrument input slit determined in ground calibrations.
$\Omega\left(\phi \right)$
is the quantity of solid angle viewed by the specific angular bin being calculated, also determined during ground calibration.
$F_{\lambda}\left(\phi,t\right)$
is the flat field correction value for the bin, interpolated to the time of the exposure. This correction is derived in orbit through nadir pointing. Each spectal line will have a different flat field profile. The average of $F_{\lambda}(\phi,t)$ is defined to be 1.0, therefore any change in efficiency over time must be captured in the $\epsilon (\lambda,t)$ term (see Sect. 5.2). This term also captures any variations in the angular scale of a pixel, including those that might be caused by differential non-linearity in the detector electronics.
$n\left(\phi,t\right)$
is the number of counts detected in the bin.
$b\left(\phi ,t\right) \frac{F_{b}\left(\phi,t\right)}{F_{\lambda}\left(\phi ,t\right)}$
is the number of counts in the same altitude bin of the background region corrected for relative flat field effects. The primary sources of background are radioactivity in the microchannel plates and charged particles entering the instrument slit. In certain observing geometries, scattered light can become a significant effect, however this scattering is limited to certain detector regions and can be detected. When we detect such scattering we exclude the data from further processing to avoid contaminated data from being used for iononspheric inversions.
$t_{\mathrm{exp}}$
is the exposure time recorded by the instrument control package. For normal observations this is 12.000 seconds.
$d(t)$
is the correction for deadtime in the detector subsystem. It is measured to ∼0.8% accuracy in each 12 second exposure through production of stimulated events ∼10 times each second.

## Ground Calibrations

Pre-flight ground calibrations were performed using this strategy:

1. We separately characterized the performance of the diffraction grating (coating efficiency, order efficiencies) and the detector quantum efficiency (QE) at discrete wavelengths.
2. For the integrated instrument, we determined the absolute throughput, wavelength scale, resolution, field of view, and in-band, and out-of-band scattering levels at discrete wavelengths.
3. We then use efficiency models provided by the diffraction grating and optical coating manufactures that are scaled to SSL measurements to predict performance at other wavelengths.

All ground calibrations were performed at SSL and are detailed in Sirk et al. (2017), Ishikawa et al. (2016).

### In-Orbit Checkout

The ICON observatory was launched into low-earth orbit on 10 October 2019. During ascent, the instrument was evacuated through low pressure poppet valves. To prevent cross contamination, the instrument vacuum door remained closed with the instrument at ∼50 torr until the other instruments were brought to their operational states. Instrument low voltage power was applied 8 days after launch on 18 October 2019. The vacuum door was opened on 2 November 2019. MCP high voltage was increased to the initial operating voltage of 4200 V between 12 November 2019 and 14 November 2019.

## In-Flight Calibrations

Since being put into operation, *ICON EUV* has undergone changes in its spectral response. Impacts include contamination and sensitivity loss due to charge depletion of the microchannel plates in regions of high count rate, primarily near the peak intensity of 83.4 nm. Such changes in instrument response could disrupt the determination of peak ion density and the altitude of peak density. To account for this effect we perform two types of in-flight calibrations, lunar calibration which provides an efficiency versus wavelength measurement (referenced to SDO EVE) at 7 vertical imaging angles, and a flat field calibration that allows us to determine the efficiency between the lunar calibration angles on a pixel by pixel basis.

### Lunar Calibrations

To determine our absolute photometric response at the science wavelengths we perform a monthly recalibration against the full moon. The EUV field of view is swept across the full moon at seven evenly spaced imaging angles ($2.5^{\circ}$ apart) at a rate of $0.25^{\circ }$ s^−1^. The lunar spectrum subtends less than 3 vertical pixels on the detector. Using the initial pre-flight calibration of the spectrometer as our starting point, and near-concurrent EUV solar measurements from the EVE instrument aboard SDO (Pesnell et al. 2012), the lunar phase function of Flynn et al. (1998), and the pre-flight spectral response in regions of the detector that are unaffected by high-rate related charge depletion, we determine the geometric EUV albedo for the moon (which does not change from month to month) and calculate the spectral response in the science regions, so as to track in response changes over time. These changes could be due to contamination from off-gassing material on the spacecraft or due to charge depletion in the microchannel plates, with in orbit charge depletion being the largest contributor to degradation.

**Fig. 3:**
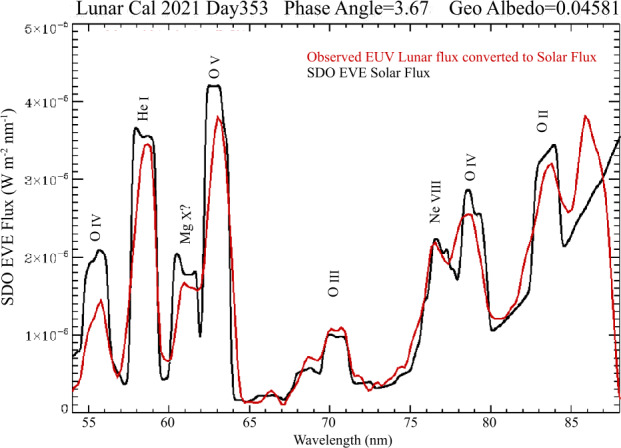
Comparison of the SDO EVE Solar spectrum and the scaled *ICON EUV* Lunar spectrum converted to solar flux, as performed in the lunar calibration process. The changes in the instrument responsivity are derived in this process. Discrepencies below 56 nm and above 85 nm are largely due to detector edge effects although some of the discrepancy below 56 nm could be due to uncertainty in where the exact edge of the rapid decline in albedo below 58 nm is located. We will be exploring this in Sirk et al. (2023)

Because this measurement is sparse, only being performed at 7 off axis angles ($\phi$), the average of these measurements of $\epsilon$ may not be reflective of the overall responsivity. The measurements at each $\phi$ must be corrected for flat field response to derive an accurate average $\epsilon(\lambda,t)$. In the calibration process we convert the EUV spectrum into a solar flux spectrum of the same units utilized by SDE EVE. This process utilizes the lunar albedo and phase function of Flynn et al. (1998). The resulting spectrum corrected for the relative flat field at the measurement angles and scaled to the EVE flux to derive a correction to average responsivity at each science wavelength relative to the first lunar calibration, which is assumed to have the same responsivity as the prelaunch measurements. One such spectrum, scaled by a single value at all wavelengths, is shown in Fig. 3.

Uncertainty in the absolute response at launch was 13%. Absolute uncertainty determination through use of SDO EVE spectra is higher as the absolute calibration determination of EVE is $\sim30$% (Chamberlin 2020, priv. commun.) over our passband. Even though our flux determinations from the first lunar calibration were significantly closer to the EVE measurement (≪15%) than would be expected given a 30% absolute error, we continue to reference our initial orbit responsivity calibration to our ground calibration. Full details of the determination of the lunar albedo and phase function will be found in reference (Sirk et al. 2023).

### Line Profile Flat Field

The second calibration is a monthly flat-field calibration. For these calibrations the spacecraft is oriented so that the EUV instrument boresight is pointing towards nadir with the slit oriented parallel to the spacecraft velocity vector. In this configuration any variation in the atmospheric radiance will quickly traverse along the slit. After a several hundred second exposure, each pixel will have seen essentially the same emitting regions and the resulting image can be used to determine a flat field correction at each of the science wavelengths at all of the imaging angles. The primary purpose of the flat field calibration is to correct for sensitivity variations in the imaging directions that could distort the determination of the altitude profile of the emission.

**Fig. 4:**
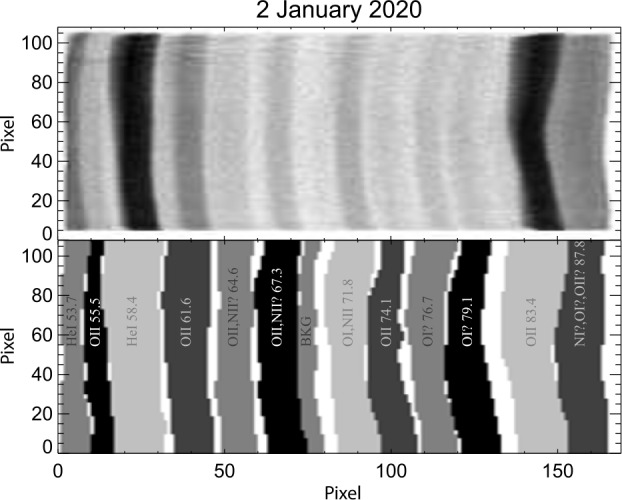
An early mission nadir calibration. The upper frame shows the nadir image. The lower frame shows the image mask for the 12 wavelength regions and the background region. The listed species and wavelength (in nm) show the expected dominant emission in each region. At this time, only the Oii 83.4 nm and 61.6 nm altitude profiles are utilized in the inversions to determine ion densities in later pipeline stages

The upper panel of Fig. 4 shows a flat field image from early in the mission. The locations and boundaries of spectral features were used to define masks for an altitude profile extraction regions for each emission feature and a background region. We determine this flat field on a “per spectral line” basis. The spectral line region is extracted using the region mask, the emission is summed along the spectral direction, and normalized to an average value of 1.0 to arrive at the flat field correction $F_{\lambda}(\phi,t)$. Because the same image mask is used for the flat field determination and for the altitude profile extraction, changes in the width of the mask at differing imaging angles are automatically corrected.

### Alignment and Pointing

The center of the field of view is defined by determining the edges of the diffraction grating field of view. The angular center of these edges in the spectral direction defines the angular offset in that direction. The edges in the imaging direction determine the imaging offset and a pixel defining the imaging center. In ground calibration this center was referenced to a reflective alignment cube mounted on the instrument. After the instrument was mounted to the spacecraft, this alignment cube was used to determine the alignment of the instrument to the spacecraft axes as defined by the attitude control system, with an estimated error of $0.05^{\circ}$ in the imaging direction.

In flight this pointing solution is confirmed during lunar calibrations. This in-flight verification has an estimated error of $0.07^{\circ}$ which corresponds to about 6 km at a 200 km tangent altitude.

### Field of View

The ultimate response of the instrument at a given wavelength is the product of the instrument throughput efficiency and the etendue (the product of the entrance aperture area and the solid angle of the sky visible by the grating). The slit dimensions were measured with a microscope on a fine micrometer stage and are 0.904 mm wide, and 40.0 mm long. The geometrical area is $0.3616 \pm0.0016$ cm^2^. The angular field of view was determined by shining a pencil beam of 83.4 nmEUV light at 3 different positions along the slit while rotating the instrument in both pitch and yaw by known amounts. The field of view measured at FWHM is 17$\, .\!\!\!^{\circ}$31 ±0$\,.\!\!\!^{\circ}$1 and 12$\,.\!\!\!^{\circ}$12 ±0$\,.\!\!\!^{\circ}$05 in the imaging and spectral directions, respectively. These values correspond to a solid angle of 0.06391 ±0.00045 sr. The lunar calibrations confirm the solid angle measurement, but are of significantly higher relative error, therefore we utilize the ground based measurements as the starting point for our in-flight calibrations.

## Post-Launch Instrument Performance Changes

By far the largest post-launch change in the function of the instrument was due to charge depletion in the pores of the microchannel plates. The channels microchannel detector operate as photomultipliers. Each event removes $10^{8-9}$ electrons from the walls of the channel. Repeated events scrub electrons from the channel walls resulting in an increase in the work function. This results in fewer electrons being emitted in subsequent events, which is seen as lower event gain (Wiza 1979). This effectively limits the lifetime of microchannel plate detectors.

**Fig. 5:**
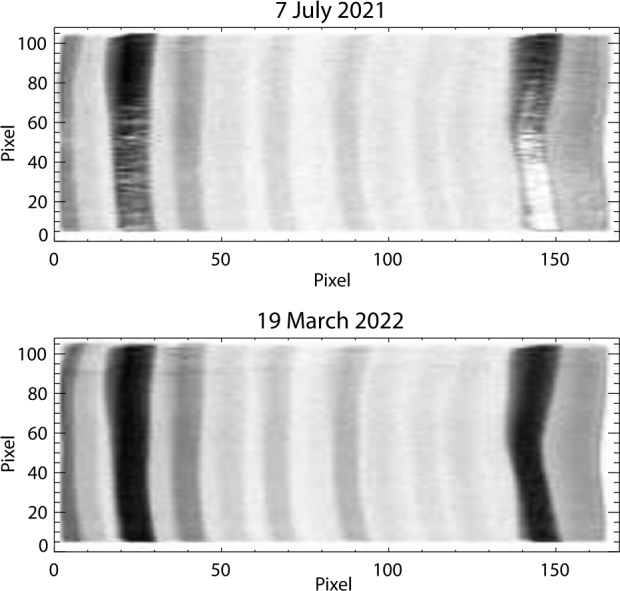
Top: A detector image from July 2021 showing significant count loss from charge depletion in the Oi 83.4 and Hei 58.4 lines. Bottom: Increasing the HV has restored counts to the depleted region

**Fig. 6:**
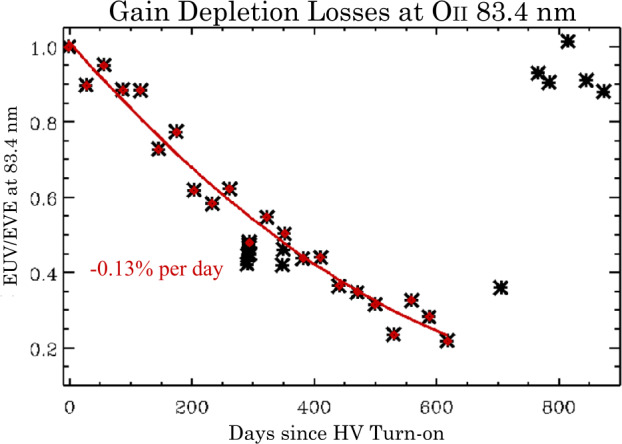
Changes in relative responsivity of *ICON EUV* due to charge depletion. Responsivity fully recovered when the HV was increased

As the gain is reduced, the charge of individual events is reduced. If it is reduced enough, some events fall below the charge threshold of the detector electronics and the effective efficiency of the detector system is reduced. It was known that charge depletion would be seen in the EUV detector, and that was part of the motivation for the flat field correction described above. If count loss becomes significant enough, it can be difficult for the flat field correction to recover the altitude profile of the affected line. A solution to this is to increase the detector gain by raising the high voltage. Figure 5 shows an image taken by the detector in July of 2021. It shows significant loss of events in the low altitude portions of Oi 83.4 nm line and Hei 58.4 nm line. The lower panel shows the spectrum in March 2022 after an increase in the high voltage. In July 2021, the flat field correction in the depleted region of 83.4 was as high as a factor of 8, which results in a large increase in the errors relative to earlier in the mission. The HV increase returned the correction to near 1 across the entire profile. The change in effective responsivity is shown in Fig. 6.

## Line Profile Processing

1. The image is shifted and/or scaled using the stim-pulser stimulation event position. Stimulation events occur 19.0735 times each second divided between the two positions. If shifts in the electronics or timing have occurred they will be reflected in the location of these events. In general no correction has been required for the vast majority of images captured.
2. A dead time correction ($d(t)$)is used to adjust the exposure time. Because the stim-pulser event occur 19.0735 times per second with high accuracy and are subject to the same dead-time as photon events, they can be used to accurately determine the deadtime. Dead-time corrections are of order 1 or 2% for daytime data, although values as high as 5% occur at times of high count rate. The dead-time correction is applied to the exposure time. For example a 12 duration second exposure, is effectively a 11.88 second exposure at 1% dead time.
3. The image is examined for excess counts in a region known to be affected by noise near the South Atlantic Anomaly. If excess counts are detected, a scaled SAA count image is subtracted from the image.
4. The image is examined for excess counts in a region that is affected by scattered solar radiation when the field of view is near the sun. Typically this occurs when the spacecraft is near the terminator at high latitude at certain times of the year. The amount of solar scattering is quantified and recorded with every 12 second observation.
5. Each region of interest is isolated by using the appropriate mask. The image is summed along the spectral direction to generate a one dimensional profile
6. The background is determined from the background region. Because both foreground and background events are affected by lower gain in the charge depleted region, the background is multiplied by flat field in the line region to appropriately model the shape of the background count profile. This is also summed in the spectral direction to create a 1-D profile. A single background profile ($b(\phi,t)$) is used for every line, although the flat field ($\frac{F_{b}(\phi,t)}{F_{\lambda}(\phi ,t)}$) applied to that profile varies from line to line. In areas of low signal (high altitude) this may lead to slight over or under subtraction, which is often an issue where subtracting numbers with less than 1$\sigma$ significance relative to the errors. This might be corrected by presuming a asymptote of zero emission for the faint lines at high altitude. As this offset would be small, it is unlikely to affect ionospheric retrievals which are dominated by brighter emission. This background subtraction was optimized for the Oii 61.7 nm line which, with Oii 83.4 nm, is a primary science line. The fainter oxygen lines have lower SNR in the flat field correction. This may result in under or oversubtraction of the background in these lines. This is a possible cause of the excess emission seen in these lines at high latitude where the emission is faint. We are considering adding this as an addition systematic error term in future software releases.
7. Flat fields from nadir calibrations bracketing the exposure in time are used to create a relative flat field. This relative flat field is adjusted to match the instrument response interpolated between bracketing lunar calibrations, creating a 1-D absolute responsivity flat field.
8. The background profile is subtracted from the foreground. This difference is divided by the dead-time corrected exposure. The difference is then multiplied by the flat field correction to generate the absolute photon rate. This is divided by the instrument area×solid-angle product to obtain the radiance.

**Fig. 7:**
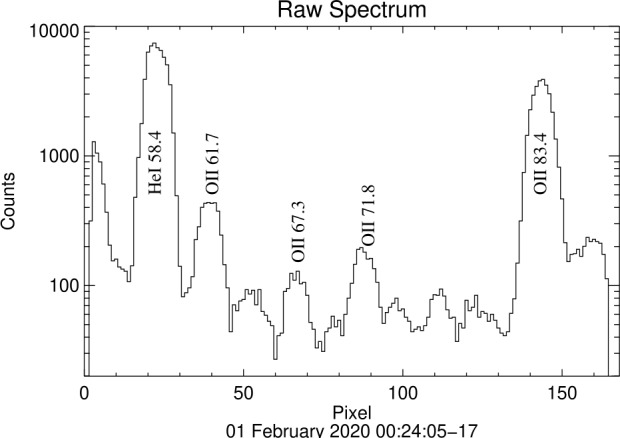
A single 12-second spectrum from *ICON EUV*

**Fig. 8:**
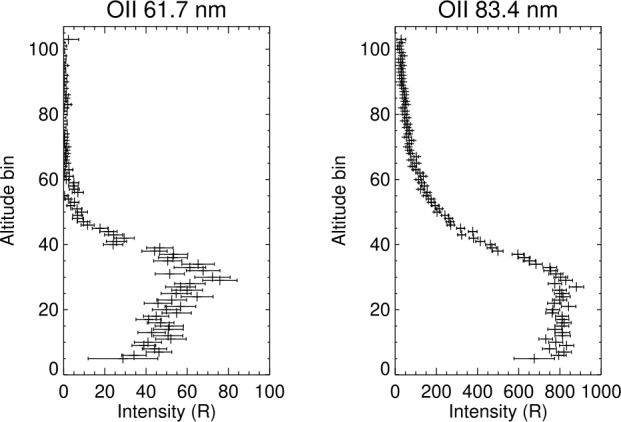
Altitude profiles for OII 61.6 nm and OII 83.4 emission using the same 12 seconds of data as in Fig. 7. The errors shown are the errors due to count statistics. Systematic errors in the absolute calibration are approximately 20%. This error effects all measurements at a given wavelength in an identical manner and does not increase the relative bin to bin errors. Conversion of these profiles to $O^{+}$ density vs altitude profiles are discussed in Stephan et al. (2022), this volume

Fig. 7 shows a single 12 second spectrum summed along the vertical dimension of the detector. This shows the dynamic range of the detected lines. Figure 8 shows altitude profiles of Oii 61.7 nm and Oii 83.4 nm emission, showing typical per bin errors. The bin spacing corresponds to 6.5 km near the emission peak.

## Conclusions

*ICON EUV* has met all of its science requirements for the 24-month baseline mission, December 2019 through February 2021. No sign of contamination after the ground calibration has been seen, which is unusual in EUV instruments. In late March of 2021, the SNR of the altitude profiles had been reduced enough that the inversions to determine O^+^ density and peak altitude were failing. This prompted the increase in detector HV to recover the signal. Of course, if operations were kept the same we would anticipate that the depletion would again become problematic in about two years of an extended mission. To avoid this we have reduced operations to the most scientifically productive regions of the orbit, near midday and near the magnetic equator. Inversions near the terminators and at high latitude have been of limited value because line of sight changes and solar interference. With these changes we expect up to 5 years of operation without being significantly affected by charge depletion.

The combination of lunar and nadir calibrations have been very successful in tracking changes in the instrument sensitivity and recovering the altitude profiles. This calibration was very necessary to calibrate the change in instrument response following the high voltage changes.

If designing the instrument today it is probable that we would make some design changes to reduce the dynamic range difference between the Oii 61.7 and the Oii 83.4 radiance. These could include a photocathode coating or a filter mesh over the 83.4 nm region to reduce effective detector efficiency.

## Footnotes

1 Spectroscopic data for this study is derived, in part, from the NIST Atomic Spectra Database (Kramida et al. 2021).
